# Effects of a ready‐to‐eat cereal formula powder on glucose metabolism, inflammation, and gut microbiota in diabetic db/db mice

**DOI:** 10.1002/fsn3.1761

**Published:** 2020-06-29

**Authors:** Caina Li, Xing Wang, Sujuan Sun, Shuainan Liu, Yi Huan, Rongcui Li, Quan Liu, Hui Cao, Tian Zhou, Lei Lei, Minzhi Liu, Zhufang Shen

**Affiliations:** ^1^ State Key Laboratory of Bioactive Substances and Functions of Natural Medicines Key Laboratory of Polymorphic Drugs of Beijing Institute of Materia Medica Chinese Academy of Medical Sciences and Peking Union Medical College Beijing China

**Keywords:** cereal formula powder, fecal metabolome, glucose metabolism, gut microbiota, inflammation

## Abstract

The cereal formula powder, Zhengda Jingshan (ZDJS), comprises dietary fiber, multivitamins, fine protein, and various cereal ingredients. The present study evaluated the effects of ZDJS on glucose metabolism and explored the corresponding mechanisms in terms of modulating gut microbiota and the fecal metabolome. Type 2 diabetic db/db mice were given ZDJS (1 g/kg) orally twice daily for 55 days, after which glucose metabolism, inflammation, gut microbiota, and fecal metabolomics were assayed. Repeated administration of ZDJS was associated with a trend toward decreasing fasting blood glucose and a 0.12% decrease in hemoglobin A1c (HbA1c), as well as statistically significant increases in the insulin sensitivity index and decreases in serum levels of tumor necrosis factor (TNF‐α) and ileum expression of mucin‐2. ZDJS also ameliorated the compensatory enlargement of islets and decreased the ratio of the α‐cell area to total islet area; however, this amelioration of impaired oral glucose tolerance became less pronounced as treatment continued. In addition, ZDJS remarkably decreased the abundance of phylum Proteobacteria and the phylum ratio of Firmicutes to Bacteroidetes, as well as altered the fecal metabolic profile. Taken together, our findings demonstrate that ZDJS improved glucose metabolism and reduced inflammation in type 2 diabetic db/db mice, which may be associated with a reshaping of the gut microbiome and fecal metabolome in db/db mice. Thus, our study suggests that ZDJS may represent a complementary therapy for patients with type 2 diabetes.

## Graphical abstract

The ready‐to‐eat cereal formula powder, ZDJS, improved glycometabolism, ameliorated inflammation, and reshaped gut microbiota and fecal metabolome in diabetic db/db mice, suggesting a complementary therapy for patients with type 2 diabetes mellitus.

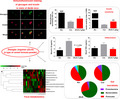

## 1 INTRODUCTION

Type 2 diabetes mellitus (T2DM) is threatening the health of more and more individuals over time. In addition, many others are prediabetic—characterized by obesity, insulin resistance, and impaired glucose tolerance—which can easily develop into overt diabetes. Although there has been an increasing number of antidiabetic drugs in the clinic, dietary and lifestyle modifications are still the most promising, safe, and economically efficient methods (Russell et al., 2016) for ameliorating symptoms, especially for individuals with prediabetes. Dietary fibers are defined as edible parts of plants or analogous carbohydrates that are resistant to digestion and absorption in the human small intestine, and with complete or partial fermentation in the large intestine (Howlett et al., 2010). Clinical studies have shown that adequate intake of dietary fiber is associated with better glycemic control, increased first‐phase insulin secretion, and improved insulin sensitivity (Bodinham, Smith, Wright, Frost, & Robertson, 2012; Fujii et al., 2013; Wolever, Campbell, Geleva, & Anderson, 2004).

Gut microbiota inhabit the distal gut and possess more carbohydrate‐active enzymes than those of eukaryotic human cells, which enable gut microbiota to depolymerize and ferment ingested food that cannot be digested in the stomach or absorbed in the intestine. Intake of dietary fiber not only influences the composition of gut microbiota (Candela et al., 2016) but also provides an essential substrate for microbiota, whereas fiber deprivation exacerbates pathogen susceptibility (Desai et al., 2016). In addition, gut microbiota ferment dietary fiber into short‐chain fatty acids (SCFAs)—such as butyrate, propionate, and acetate—which are important signaling molecules for transducing the functions of gut microbiota to the host, thus influencing appetite regulation, immune function, epithelial cell integrity, and homeostasis of glucose and lipids (Holscher, 2017; Koh, De Vadder, Kovatcheva‐Datchary, & Bäckhed, 2016).

Zhengda Jingshan (ZDJS) is a type of ready‐to‐eat cereal formula powder consisting of dietary fiber, multivitamins, fine protein, and various cereal ingredients, and the total energy of 30 g ZDJS is 421 KJ. The proportions of nutrient reference values for dietary fiber, protein, fat, and carbohydrates in ZDJS are 48%, 13%, 5%, and 2%, respectively. The detailed ingredients and contents of ZDJS are listed in Table S1. The present study aimed to evaluate the effects of ZDJS on glucose metabolism and to explore its functional mechanisms in terms of modulation of gut microbiota.

## 2 MATERIALS AND METHODS

### 2.1 Animals

Male BKS‐Lepr^em2Cd479^/Nju (Lepr KO/KO, db/db) mice and wild‐type control mice (Lepr wt/wt, db/m), aged 4–8 weeks, were obtained from the Nanjing Biochemical Research Institute of Nanjing University (Nanjing, China) and were raised with special feed (XieTong Organism, Nanjing, China) in a temperature‐ and humidity‐controlled environment with a 12‐hr light/dark cycle and free access to water. Diabetic db/db mice were divided into a vehicle‐treated group (Con) and a ZDJS‐treated group (ZDJS, 1 g/kg; *n* = 11). Age‐matched wild‐type mice were used as healthy controls (Nor, *n* = 12). ZDJS‐treated mice were given ZDJS in water (1 g/kg, 0.1 ml/10 g BW) twice daily for 55 days. Vehicle‐treated mice and healthy control mice were given equal volumes of water. The present study was carried out with another study; these two studies used the same diabetic control mice and healthy control mice.

### 2.2 Assays measuring nonfasting blood glucose, fasting blood glucose, and HbA1c

After 10, 25, 42, and 45 days of treatments, blood was collected from tail tips to determine nonfasting blood glucose (NFBG) levels via the glucose oxidase method (Biosino Bio‐Technology & Science Inc., Beijing, China). After 10, 22, 30, and 37 days of treatments, all of the mice were fasted for 4 hr with free access to water, and then, fasting blood glucose (FBG) levels were determined via the aforementioned glucose oxidase method. After 45 days of treatments, hemoglobin A1c (HbA1c) levels were measured with a commercial kit (Homa Biological, Beijing, China).

### 2.3 Assays measuring oral glucose tolerance and insulin sensitivity

All of the mice were fasted for 4 hr, with water provided ad libitum, before the start of experiments. After 16 and 37 days of treatments, the oral glucose tolerance test (OGTT) was separately performed; the insulin tolerance test (ITT) was carried out after 30 days of treatment, as previously described (Peng et al., 2014). The area under the blood glucose–time curve (AUC) and the decreasing ratio of blood glucose at 40 min after injection of insulin were calculated in the ITT. In addition, fasting blood insulin (FBI) levels were determined (ALPCO, Salem, NH, USA) after 42 days of treatment, as was FBG. Then, the insulin sensitivity index (ISI) was calculated as follows: 1/(FBG × FBI).

### 2.4 Assays measuring serum TNF‐α, IL‐1β, and glucagon levels

After 54 days of treatments, all of the mice were fasted for 4 hr, with water provided ad libitum, followed by collection of whole blood from the orbital cavity for determination of glucagon (R&D, Minneapolis, MN, USA), tumor necrosis factor (TNF‐α), and interleukin‐1β (IL‐1β) (R&D, Minneapolis, MN, USA).

### 2.5 Immunofluorescent staining of insulin and glucagon, and immunohistochemical staining of mucin‐2 and occludin

After 55 days of treatments, all of the mice were fasted (as described above) and were sacrificed via cervical dislocation. Subsequently, the pancreas and ileum were isolated, separately fixed in Bouin and paraformaldehyde solution, and then, both were embedded in paraffin. Serial 5‐µm sections of each collected pancreas were prepared for staining of insulin and glucagon (*n* = 6), and sections of ileum were stained for mucin‐2 and occludin (*n* = 4) (Li et al., 2019; Li et al., 2017).

### 2.6 Gut microbial sequencing and fecal metabolomic analysis

After 44 days of treatments, feces were collected and subjected to gut microbial sequencing and metabolomic analysis, which were performed and analyzed as previously described (Li et al., 2019).

### 2.7 Statistical analysis

Data are expressed as the mean ± standard error of the mean (*SEM*) and were analyzed using either two‐tailed Student's *t* tests or one‐way analyses of variance (ANOVAs) with Bonferroni corrections via GraphPad Prism 8.01 (GraphPad Software, San Diego, USA). The OGTT and ITT data were analyzed with two‐way ANOVAs with Bonferroni corrections. A *p* < .05 was considered to be statistically significant.

## 3 RESULTS

### 3.1 Effects of ZDJS on glycemic control and insulin sensitivity

**FIGURE 1:**
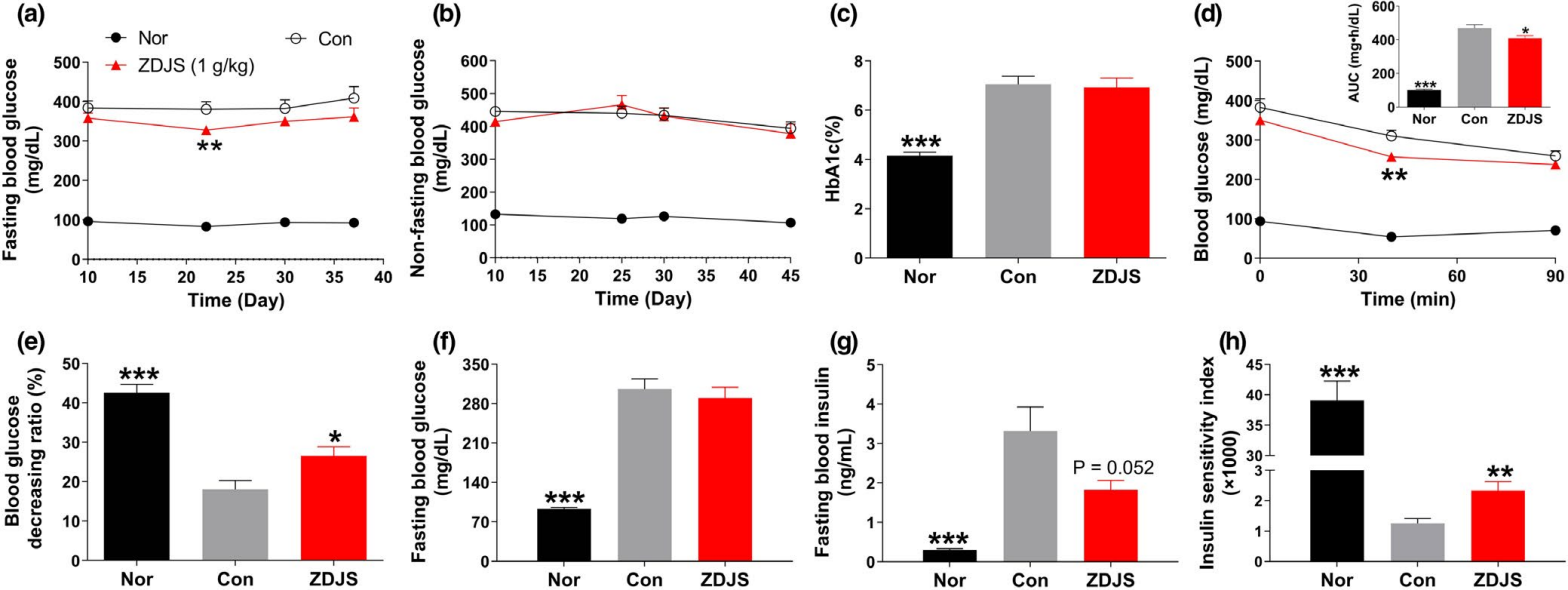
Effects of ZDJS on glycaemia and insulin sensitivity in diabetic db/db mice. (a) Fasting blood glucose, (b) nonfasting blood glucose, and (c) HbA1c are shown. (d) Insulin tolerance test was performed after treatments for 30 days, and the blood glucose variation and area under the blood glucose–time curve (AUC) are shown. (e) The decreasing ratio of blood glucose at 40 min after subcutaneous injection of insulin is shown. (f–i) Fasting blood glucose (f), fasting blood insulin (g), and the insulin sensitivity index (h) after treatment for 42 days are shown. Data are expressed as the mean ± *SEM* (*n* = 9–12, ***p < .001, **p < .01 vs. Con). Nor denotes the healthy control mice; Con denotes diabetic mice; and ZDJS denotes Zhengda‐Jingshan‐treated mice

In comparison with those of the Con group, repeated treatments with ZDJS (1 g/kg) had no significant influences on FBG or NFBG (Figure 1a and b), but it decreased FBG by 6.71% (*p* > .05), 13.83% (*p* < .05), 8.52% (*p* > .05), and 11.60% (*p* > .05) after 10, 22, 30, and 37 days of treatment, respectively. These values were calculated using the average blood glucose levels. In addition, repeated treatments with ZDJS reduced the HbA1c level by 0.12% but the difference was not statistically significant (Figure 1c). Repeated treatments with ZDJS for 30 days significantly decreased the AUC and blood glucose decreasing ratio after 40 min of insulin injection (vs. the blood glucose level at 0 min) in the ITT (Figure 1d and e). Furthermore, repeated treatments with ZDJS for 42 days did not significantly alter FBI levels (*p* = .052, Figure 1g) but did elevate the ISI (*p* < .01, Figure 1h).

### 3.2 Effects of ZDJS on β‐cell function and the α‐ and β‐cell ratio in islets

**FIGURE 2:**
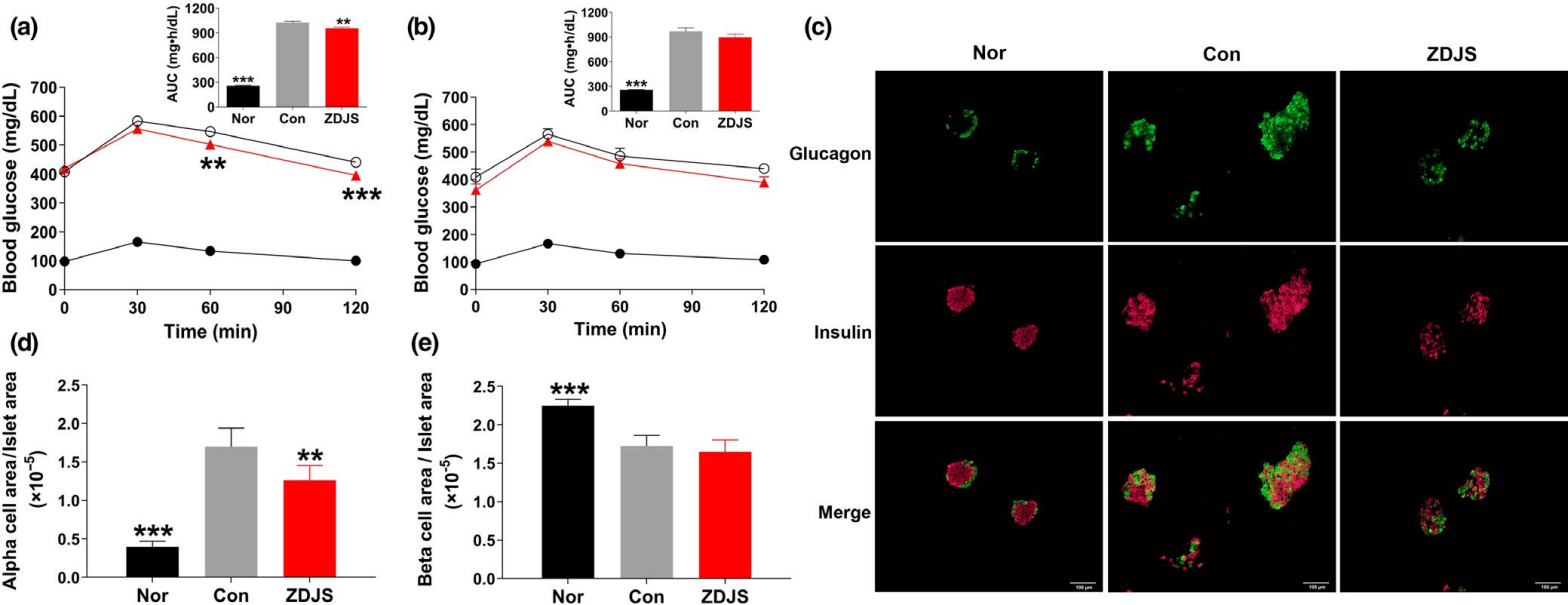
Effects of ZDJS on β‐cell function and the α‐ and β‐cell ratio in islets. (a–b) The oral glucose tolerance test was performed after treatments for 16 days (a) and 37 days (b), and the blood glucose variation and area under the blood glucose–time curve (AUC) are shown. (c) Immunofluorescent staining of glucagon and insulin is shown. Images were obtained at 200× magnification. (d–e) Analysis of the ratios of the α‐cell area to total islet area (d) and the β‐cell area to total islet area (e). Data are expressed as the mean ± *SEM* (*n* = 10–12 for a and b, *n* = 6 for c–e. ***p < .001, **p < .01 vs. Con). Nor denotes the healthy control mice; Con denotes diabetic mice; and ZDJS denotes Zhengda‐Jingshan‐treated mice

In comparison with those of the Con group, repeated treatments with ZDJS (1 g/kg) decreased blood glucose levels and AUC following oral glucose loading after treatment for 16 days (*p* < .05, Figure 2a), but this was attenuated after 37 days of treatment (Figure 2b). In addition, immunofluorescent staining showed that repeated treatments with ZDJS ameliorated the compensatory increase of islets in the Con group (Figure 2c) and reduced the ratio of the α‐cell area to total islet area (*p* < .01, Figure 2d), which was consistent with the reduction of fasting blood glucagon (31.7 ± 2.9 for ZDJS vs. 65.0 ± 11.3 for Con), but did not influence the distributions of α cells or β cells (Figure 2c) or the ratio of the β‐cell area to total islet area (Figure 2e).

### 3.3 Effects of ZDJS on intestinal mucin‐2 expression and serum TNF‐α and IL‐1β levels

**FIGURE 3:**
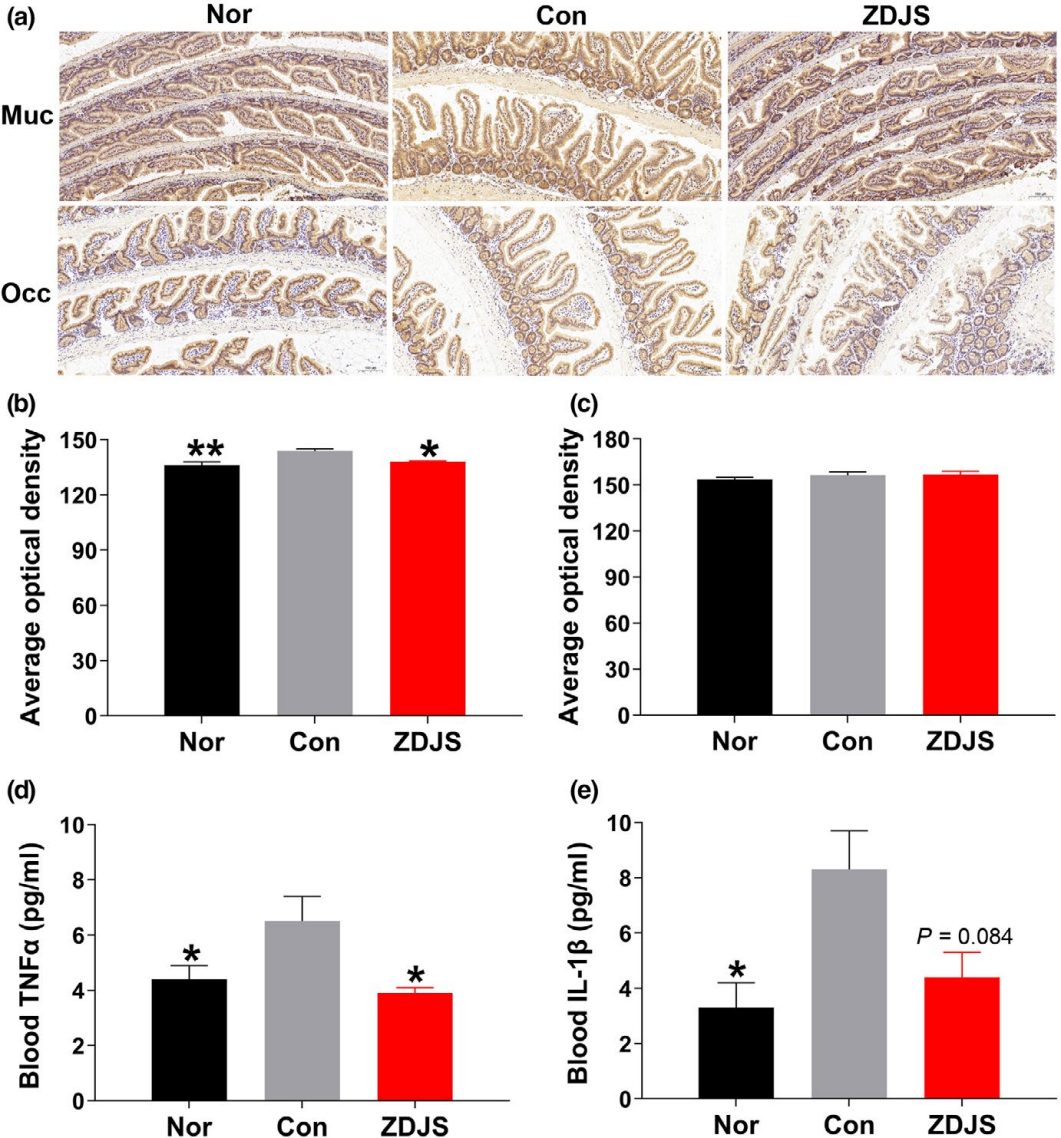
Effects of ZDJS on intestinal mucin‐2 expression and serum TNF‐α and IL‐1β levels (a) Immunohistochemical staining of mucin‐2 and occludin is shown. Images were obtained at 200× magnification. (b–c) Average optical‐density analysis of mucin‐2 (b) and occludin (c) is shown. (d) Blood TNF‐α is shown. (e) Blood IL‐1β is shown. Data are expressed as the mean ± *SEM* (*n* = 4 for a–c, 8–12 for d and *n* = 6–10 for e. **p < .01, *p < .05 vs. Con). Nor denotes the healthy control mice; Con denotes diabetic mice; and ZDJS denotes Zhengda‐Jingshan‐treated mice

In comparison with those of Nor, intestinal mucin‐2 expression and serum TNF‐α and IL‐1β levels were significantly increased in diabetic db/db mice (Con), and repeated treatments with ZDJS remarkably decreased the expression of mucin‐2 (Figure 3a and b) and lowered serum TNF‐α (*p* < .05), but they had no significant influence on IL‐1β (*p* = .064) levels (Figure 3d and e). There were no differences in the expression levels of occludin among the three groups (Figure 3a and c).

### 3.4 Effects of ZDJS on gut microbiota profiles

**FIGURE 4:**
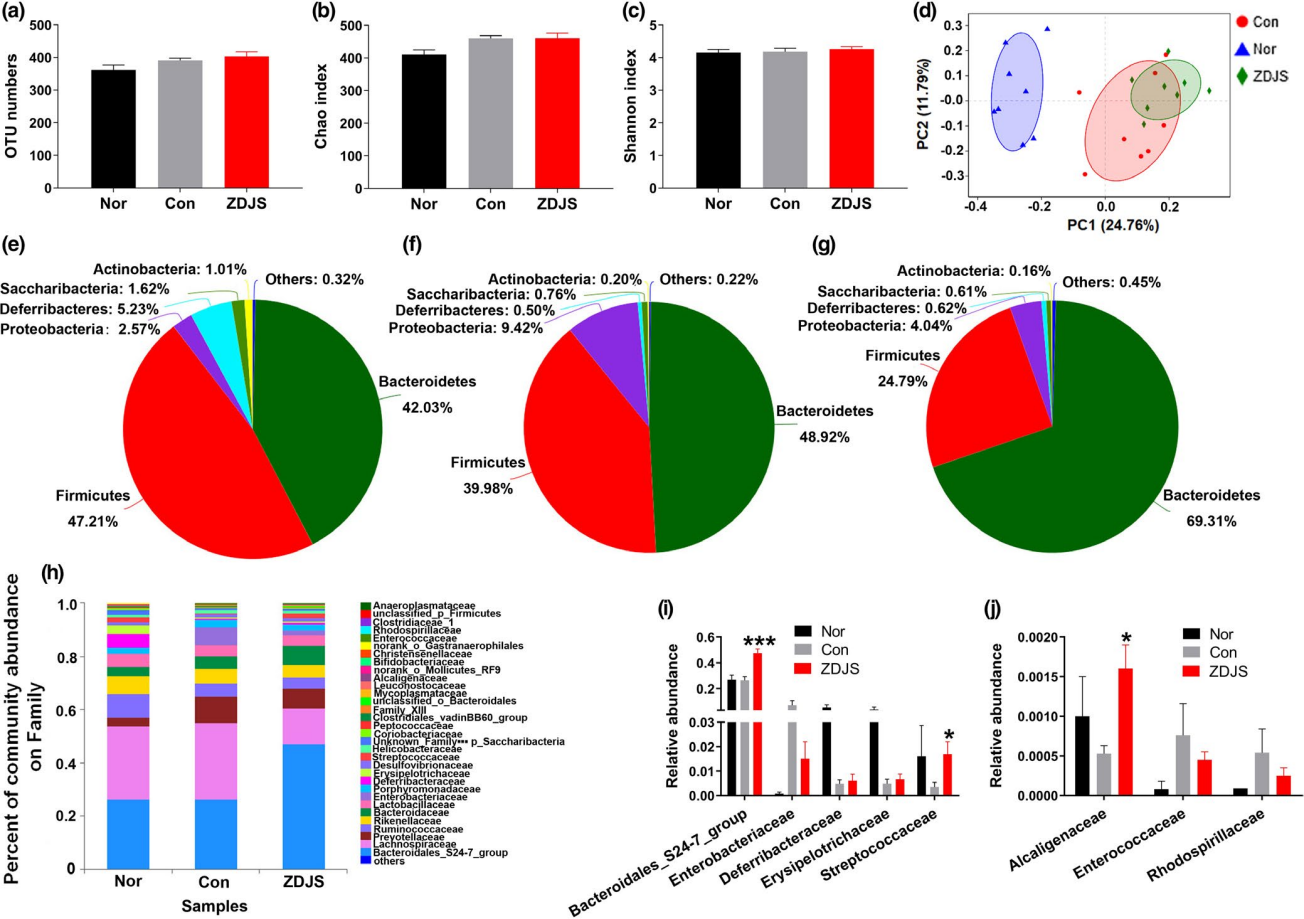
Effects of ZDJS on gut microbiota profiles. (a) Total OTU numbers, (b) Chao index, (c) Shannon index, and (d) PCoA based on Bray‐Curtis are shown. e–g) Pie charts of the relative abundances of bacteria at the phylum level in the Nor (e), Con (f), and ZDJS (g) groups are shown. (h) Column graphs of the relative abundances of bacteria at the family level are shown. (i–j) The relative abundances of specific bacteria at the family level are shown. Data for a–c are expressed as the mean ± *SEM* (*n* = 8). Nor denotes the healthy control mice; Con denotes diabetic mice; and ZDJS denotes Zhengda‐Jingshan‐treated mice

Illumina sequencing produced a total of 704,904 sequences from 24 samples after normalization based on the minimum sample‐sequence number, and these sequences were then clustered into 665 operational taxonomic units (OTUs, Table S2). Based on the analysis of OTUs, the OTU numbers and the Chao index or Shannon index were not significantly different among the three groups (Figure 4a–c). However, principal coordinates analysis (PCoA) based on the Bray–Curtis showed significant variation between the Nor and Con groups, whereas ZDJS diverged from the Con group along the first principal coordinate (PC1) and the second principal coordinate (PC2) (Figure 4d).

In comparison with that of Nor, diabetic db/db mice displayed disparate community abundance, but all groups possessed dominant bacterial phyla in terms of Firmicutes and Bacteroidetes (Figure 4e–g). In comparison with the results of the Con group, the ZDJS‐treated group exhibited a decreased ratio of Firmicutes to Bacteroidetes and a reduced abundance of Proteobacteria (Figure 4f and g). In addition, the ZDJS‐treated group also exhibited a different bacterial community from that of the Con group at the family level (Figure 4h) and also had a significantly increased relative abundance of *Bacteroidales_S24‐7_group* (*p* < .001)*, Streptococcaceae* (*p* < .05), and *Alcaligenaceae* (*p* < .05) (Figure 4i and j).

### 3.5 Effects of ZDJS on the fecal metabolome

**FIGURE 5:**
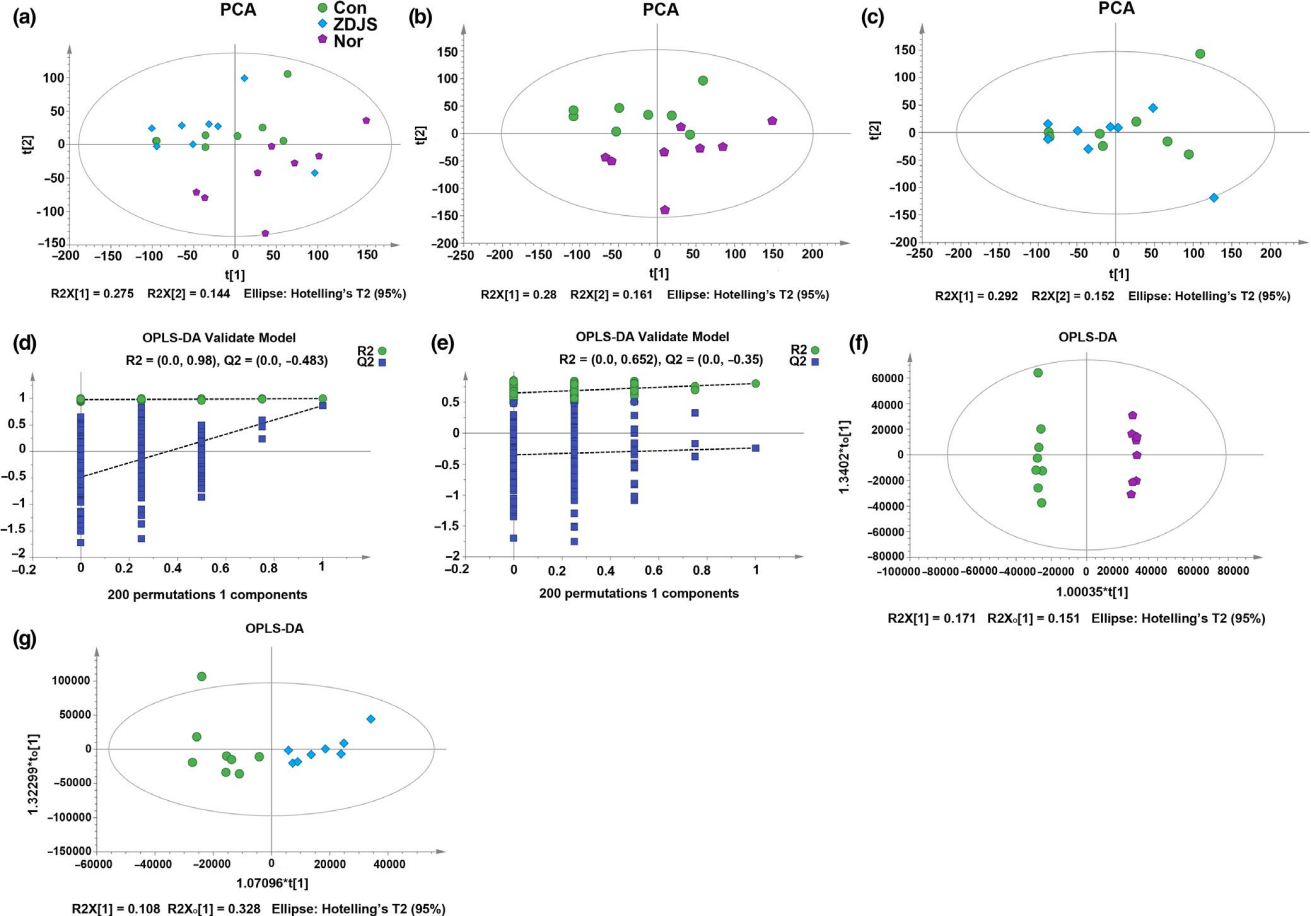
Metabolomic characteristics in feces. (a) PCA from all samples is shown. (b–c) PCA for the comparisons of the Nor versus Con groups (b) and for those of the Con versus ZDJS groups (c) are shown. (d–e) Permutation tests for the comparisons of the Nor versus Con groups (d) and the Con versus ZDJS groups (e) are shown. (f–g) OPLS‐DA for the comparisons of the Nor versus Con groups (f) and Con versus ZDJS groups (g) are shown (*n* = 8). Nor denotes the healthy control mice; Con denotes diabetic mice; and ZDJS denotes Zhengda‐Jingshan‐treated mice

Principal component analysis (PCA) revealed that there were no outlier data from any samples and comparisons between the Nor and Con groups (Figure 5a and b), but one outlier was found following a comparison of the Con group and ZDJS group (Figure 5c). In addition, orthogonal partial least‐squares discriminant analysis (OPLS‐DA) was verified by the permutation test, which showed that it was not random or overfitted in the comparisons of Nor versus Con groups or the Con versus ZDJS groups (Figure 5d and e). The OPLS‐DA results revealed that there were significant differences in the comparisons of Nor versus Con groups or the Con versus ZDJS groups (Figure 5f and g).

**FIGURE 6:**
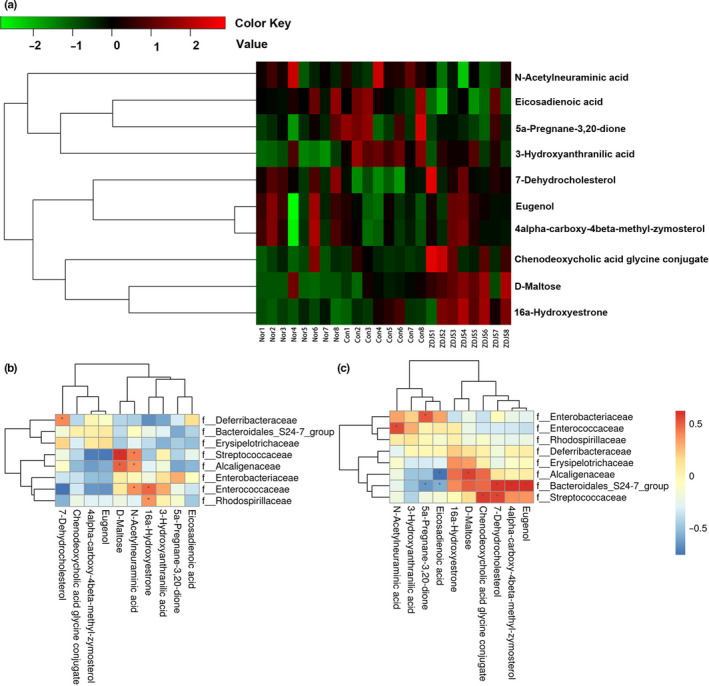
Differential metabolites and correlation analysis of gut microbiota. (a) Heat maps of the ten differential metabolites between diabetic db/db mice treated with ZDJS and vehicle are shown. (b–c) Correlation analysis for the comparisons of Nor versus Con groups (b) and Con versus ZDJS groups (c) (*n* = 8). Nor denotes the healthy control mice; Con denotes diabetic mice; and ZDJS denotes Zhengda‐Jingshan‐treated mice

A total of 60 differential metabolites were screened between the Con group and the ZDJS‐treated group, and ten of the differential metabolites—including 16‐hydroxyestrone, 3‐hydroxyanthranilic acid, 4α‐carboxy‐4β‐methyl‐zymosterol, 5a‐pregnane‐3,20‐dione, 7‐dehydrocholesterol, chenodeoxycholic acid glycine conjugate, D‐maltose, eicosadienoic acid, eugenol, and N‐acetylneuraminic acid—were classified into 21 pathways (Table S3, Table 1). In comparison with those of the Con group, repeated treatments with ZDJS significantly increased the metabolites, namely 4α‐carboxy‐4β‐methyl‐zymosterol, eugenol, 7‐dehydrocholesterol, chenodeoxycholic acid glycine, D‐maltose, and 16a‐hydroxyestrone, and decreased the metabolites, namely 5a‐pregnane‐3,20‐dione, eicosadienoic acid, N‐acetylneuraminic acid, and 3‐hydroxyanthranilic acid (Figure 6a).

**TABLE 1:** Ten differential metabolites in the comparison of Con versus ZDJS

KEGG/HMDB/LMID	Metabolites	Class	Pathway
C05300	16a‐Hydroxyestrone	Steroids and steroid derivatives	Steroid hormone biosynthesis
HMDB00335			
C00632	3‐Hydroxyanthranilic acid	Benzene and substituted derivatives	Aminobenzoate degradation Tryptophan metabolism
HMDB01476			
C15808	4alpha‐carboxy‐4beta‐methyl‐zymosterol	Sterol lipids	Biosynthesis of antibiotics Steroid biosynthesis
LMST01010150			
C03681	5a‐Pregnane‐3,20‐dione	Steroids and steroid derivatives	Steroid hormone biosynthesis
HMDB03759			
C01164	7‐Dehydrocholesterol	Steroids and steroid derivatives	Rheumatoid arthritis Steroid biosynthesis Vitamin digestion and absorption
C05443			
HMDB00032			
C05466	Chenodeoxycholic acid glycine conjugate	Steroids and steroid derivatives	Bile secretion Cholesterol metabolism Primary bile acid biosynthesis Secondary bile acid biosynthesis
HMDB00637			


C00208	D‐Maltose	Organooxygen compounds	ABC transporters Bacterial chemotaxis Carbohydrate digestion and absorption Phosphotransferase system (PTS) Starch and sucrose metabolismTaste transduction
HMDB00163			




C16525	Eicosadienoic acid	Fatty acyls	Biosynthesis of unsaturated fatty acids
HMDB05060			
C10453	Eugenol	Phenols	Phenylpropanoid biosynthesis
HMDB05809			
C00270	N‐Acetylneuraminic acid	Organooxygen compounds	Amino sugar and nucleotide sugar metabolism
HMDB00230			

### 3.6 Network analysis

Correlations among the ten differential metabolites (Figure 6a) and the eight selected bacterial families were analyzed (Figure 4i and j). In the comparison of the Con versus ZDJS groups, the bacterial *Bacteroidales_S24‐7_group* was positively correlated with the metabolites, namely 4α‐carboxy‐4β‐methyl‐zymosterol, eugenol, and 7‐dehydrocholesterol, and was also negatively correlated with the metabolites, namely 5a‐pregnane‐3,20‐dione and eicosadienoic acid; the bacterial *Streptococcaceae* was positively correlated with the metabolites, namely chenodeoxycholic acid glycine and 7‐dehydrocholesterol; the bacterial *Alcaligenaceae* was positively correlated with D‐maltose and was negatively correlated with eicosadienoic acid (Figure 6b and c).

## 4 DISCUSSION

Intake of dietary fiber is efficacious in reducing the risk of T2DM and in improving insulin resistance (Weickert & Pfeiffer, 2018), especially when taken in the form of insoluble cereal fibers that are nonviscous and fermented by gut microbiota in the colon; in contrast, soluble and viscous fibers from fruit and certain vegetables are not as efficacious (Davison & Temple, 2018; Weickert & Pfeiffer, 2018). Thus, a dietary regimen along with increased physical activity is considered to be beneficial for T2DM treatment (Russell et al., 2016). However, patients with T2DM should always consult physicians about dietary supplementations if they are already taking drug treatments, because one previous report showed a high possible risk of interactions between dietary supplementation and antidiabetic drugs (Zabłocka‐Słowińska, Dzielska, Gryszkin, & Grajeta, 2014). ZDJS is a type of ready‐to‐eat cereal formula powder comprising various cereal ingredients, as well as dietary fiber, multivitamins, and fine protein. Our present study showed that ZDJS significantly increased insulin sensitivity and decreased inflammation in diabetic db/db mice.

Islet β‐cell function is a crucial determinant for the development of overt hyperglycemia, and it adapts to metabolic burden in the face of insulin resistance through increasing insulin secretion to maintain euglycemia (Boland, Rhodes, & Grimsby, 2017). However, sustained insulin demand eventually results in β‐cell loss and dysfunction, which leads to the progression of T2DM. Thus, ameliorating the burden of insulin secretion represents a means of protecting β‐cell function. Our present study found that ZDJS treatment significantly relieved the compensatory hypertrophy of islets and decreased the ratio of α‐cells to total islets, whereas it had no influence on the ratio of β cells to total islets. In addition, the OGTT is a commonly used method for evaluating β‐cell function. In our present study, although ZDJS treatment improved impaired oral glucose tolerance, this effect was attenuated over time. In contrast, ZDJS significantly increased insulin sensitivity, which suggested that ZDJS treatment ameliorated the compensatory hypertrophy of islets through improving insulin sensitivity.

T2DM is also closely associated with chronic inflammation, which contributes to insulin resistance (Lontchi‐Yimagou, Sobngwi, Matsha, & Kengne, 2013), and many studies have shown that intake of dietary fiber significantly alleviates inflammation (Awika, Rose, & Simsek, 2018; Vieira et al., 2017; Zhang et al., 2016). Our present study showed that ZDJS decreased the inflammatory cytokine TNF‐α in diabetic db/db mice. Furthermore, mucin‐2 is a large glycoprotein secreted from goblet cells, which play a crucial role in maintaining intestinal balance (Kim & Ho, 2010). One report indicated that the expression of mucin‐2 was increased under a chronic inflammatory state and that high expression of mucin‐2 induced endoplasmic reticulum stress (ERS) and apoptosis of goblet cells (Mejías‐Luque et al., 2010; Tawiah et al., 2018). Our present study demonstrated that ZDJS decreased the diabetic‐induced increased expression of mucin‐2 in the ileum of diabetic db/db mice, which was consistent with the amelioration of inflammation and suggested relief of ERS. However, a previous report showed that dietary pea fiber supplementation significantly increased the gene expression of mucin‐2 in high‐fat diet‐induced glucose‐intolerant rats (Hashemi, Fouhse, Im, Chan, & Willing, 2017), which may have been caused by the different glycometabolic and inflammatory states between animal models.

Gut microbiota are a complex community of more than 1,000 species and have been demonstrated to be indispensable for the maintenance of host health (Sommer & Bäckhed, 2013). Compositional abnormalities of gut microbiota have been shown to be strongly associated with metabolic disorders (Cox, West, & Cripps, 2015; Qin et al., 2012), and modulation of microbiota may mitigate or even reverse such disorders (Marchesi et al., 2016). A systematic review showed that increasing cereal fiber intake should be encouraged for promoting gut microbiota diversity and overall health (Houghton et al., 2018; Jefferson & Adolphus, 2019). Although the relationship between the ratio of Firmicutes to Bacteroidetes and obesity has remained controversial (Collado, Isolauri, Laitinen, & Salminen, 2008; Koliada et al., 2017), one report showed that the ratio of Firmicutes to Bacteroidetes was higher in obese men than in obese women and was inversely correlated with peripheral insulin sensitivity in men, but not in women (Most et al., 2017). In our present study, we did not find any change in the ratio of Firmicutes to Bacteroidetes in diabetic obese db/db mice compared to that in nondiabetic lean control mice, but ZDJS decreased this ratio compared to that in untreated db/db mice, which may explain the concomitantly improved insulin sensitivity.

The phylum Proteobacteria is less abundant than other phyla, such as Firmicutes and Bacteroidetes. Some reports have shown that intake of artificial sweeteners and emulsifiers that impair glucose metabolism simultaneously increases the abundance of Proteobacteria (Chassaing et al., 2015; Suez et al., 2014), suggesting a correlation between glucose metabolism and Proteobacteria. Furthermore, a bloom of Proteobacteria is considered to be a microbial signature of gut dysbiosis, which has been observed in inflammatory diseases, such as inflammatory bowel disease, colorectal cancer, and necrotizing enterocolitis (Morgan et al., 2012; Normann, Fahlén, Engstrand, & Lilja, 2013; Wang et al., 2012), as well as in diseases with low‐level inflammation (such as irritable syndrome and metabolic syndrome) (Carroll, Ringel‐Kulka, Siddle, & Ringel, 2012; Fei & Zhao, 2013). Thus, an increased abundance of Proteobacteria is proposed to represent a potential diagnostic indicator of disease (Shin, Whon, & Bae, 2015). In the present study, an increase of Proteobacteria was found in diabetic db/db mice in comparison with that in nondiabetic control mice but was significantly decreased via ZDJS, which may have contributed to the ZDJS‐induced amelioration of inflammation and insulin sensitivity.

The gut microbial profile is closely associated with the metabolome. In the present study, we also determined the variation of the fecal metabolome and found ten differential metabolites between the Con and ZDJS groups. Two of these differential metabolites, eugenol and eicosadienoic acid, are both natural compounds, and eugenol possesses significant anti‐inflammatory and antioxidant properties, whereas eicosadienoic acid has been reported to modulate the metabolism of polyunsaturated fatty acids and alter the responses of macrophages to inflammatory stimulation (Barboza, da Silva Maia Bezerra Filho, Silva, Medeiros, & de Sousa, 2018; Huang, Huang, Li, & Chuang, 2011). Another of these metabolites, N‐acetylneuraminic acid, has been reported to exacerbate inflammation through promoting the outgrowth of *Escherichia coli* during inflammation (Huang, Chassard, Hausmann, von Itzstein, & Hennet, 2015). In addition, another one of these metabolites, chenodeoxycholic acid glycine, is the physiological ligand for the farnesoid X receptor and has been approved for the treatment of patients with cerebrotendinous xanthomatosis, who develop abnormal lipid storage with increased plasma and tissue levels of cholesterol (Fiorucci & Distrutti, 2019). The differential metabolite, 3‐hydroxyanthranilic acid, is a metabolite of kynurenine and has been reported to be positively correlated to impaired glucose tolerance in patients with obesity and is significantly decreased following bariatric surgery (Christensen et al., 2018). All of these metabolites provide a basis for ZDJS‐mediated amelioration of inflammation and improvement of glucose metabolism in diabetic db/db mice. However, there have been no reports regarding the influences of the differential metabolites, 16a‐hydroxyestrone and 5a‐pregnane‐3,20‐dione, which are metabolites of estradiol and progesterone, respectively, as well as another three differential metabolites, 4α‐carboxy‐4β‐methyl‐zymosterol, 7‐dehydrocholesterol, and D‐maltose, on glycolipid metabolism.

In addition, we found a significant correlation between the differential metabolites and species of bacteria at the family level, suggesting that ZDJS may change the fecal metabolome through modulating the gut microbial profile and subsequently improving glucose metabolism and inflammatory status in diabetic db/db mice.

In summary, intake of the cereal formula powder, ZDJS, improved insulin sensitivity, ameliorated the compensatory increase of islets, and relieved inflammation in diabetic mice. In addition, ZDJS reshaped the gut microbiota profile through decreasing the ratio of the Firmicutes to Bacteroidetes phyla and the abundance of phylum Proteobacteria, as well by altering the fecal metabolome, which may shed light on mechanisms for improving glucose metabolism and ameliorating inflammation. Taken together, our findings suggest that ZDJS may represent a complementary therapy for patients with T2DM.

## 5 INFORMED CONSENT

The written informed consent was obtained from all study participants.

## CONFLICTS OF INTEREST

All of the authors declare that there are no conflicts of interest.

## AUTHOR CONTRIBUTIONS

C.L. designed the study, performed the experiments, analyzed the data, and prepared the manuscript. X. W. and S. S. participated in the insulin tolerance test and oral glucose tolerance test. The other authors took part in measuring biochemical indices or participated in the immunofluorescent and histochemical staining. Z. S. also supplied academic and technical support. All of the authors took part in collecting tissue samples at the end of the experiment.

## ETHICAL APPROVAL

All of the protocols were performed in accordance with the “3R” principles and guidelines for laboratory animals (GB14925‐2001 and MOST 2006a) established by the People's Republic of China and were approved by the Experimental Animal Welfare Ethics Committee of the Institute of Materia Medica (Chinese Academy of Medical Sciences and Peking Union Medical College) under No. 00000814.

## Supporting information

Table S1

Table S2

Table S3
