# Supplementary material for: Effects of a ready‐to‐eat cereal formula powder on glucose metabolism, inflammation, and gut microbiota in diabetic db/db mice
Source: Food Sci Nutr. 2020 Jun 29;8(8):4523–33. doi: 10.1002/fsn3.1761 (PMC7455957; doi:10.1002/fsn3.1761)
Supplement: Supplementary file 1 — Table S1 [file FSN3-8-4523-s001.docx]

**Table 1S The detailed information of ingredients and contents in one package of ZDJS (30 g)**

| **Ingredient** | **Per package (30 g)** | **NRV%** |
| --- | --- | --- |
| Energy | 421 KJ | 5% |
| Protein | 7.6 g | 13% |
| Fat | 3.0 g | 5% |
| Carbohydrate | 5.0 g | 2% |
| Dietary fiber | 12.0 g | 48% |
| Sodium | 183 mg | 9% |
| Vitamin A | 180 µgRE | 22% |
| Vitamin E | 2.40 mg α-TE | 17% |
| Vitamin B_1_ | 0.41 mg | 29% |
| Vitamin B_2_ | 0.41 mg | 29% |
| Vitamin B_6_ | 0.41 mg | 29% |
| Vitamin B_12_ | 0.20 µg | 8% |
| Nicotinic acid | 4.26 mg | 30% |
| Folic acid | 63 µg DFE | 16% |
| Calcium | 180 mg | 22% |
| Zinc | 3.35 mg | 22% |

NRV: Nutrition Reference Values
